# Appendectomy as part of Ladd’s procedure: a systematic review and survey analysis

**DOI:** 10.1007/s00383-023-05437-7

**Published:** 2023-04-03

**Authors:** Montaser Nabeeh Al Smady, Salama Bin Hendi, Sarah AlJeboury, Hessa Al Mazrooei, Hussein Naji

**Affiliations:** 1 Mohammed Bin Rashid University of Medicine and Health Sciences, Dubai, UAE; 2 Mediclinic Parkview Hospital, Dubai, UAE

**Keywords:** Ladd’s procedure, Appendectomy, Intestinal malrotation, Children

## Abstract

**Background:**

Ladd’s Procedure has been the surgical intervention of choice in the management of congenital intestinal malrotation for the past century. Historically, the procedure included performing an appendectomy to prevent future misdiagnosis of appendicitis, since the location of the appendix will be shifted to the left side of the abdomen. This study consists of two parts. A review of the available literature on appendectomy as part of Ladd’s procedure and then a survey sent to pediatric surgeons about their approach (to remove the appendix or not) while performing a Ladd’s procedure and the clinical reasoning behind their approach.

**Methods:**

The study consists of 2 parts: (1) a systematic review was performed to extract articles that fulfill the inclusion criteria; (2) a short online survey was designed and sent by email to 168 pediatric surgeons. The questions in the survey were centered on whether a surgeon performs an appendectomy as part of the Ladd’s procedure or not, as well as their reasoning behind either choice.

**Results:**

The literature search yielded five articles, the data from the available literature are inconsistent with performing appendectomy as part of Ladd's procedure. The challenge of leaving the appendix in place has been briefly described with minimal to no focus on the clinical reasoning. The survey demonstrated that 102 responses were received (60% response rate). Ninety pediatric surgeons stated performing an appendectomy as part of the procedure (88%). Only 12% of pediatric surgeons are not performing appendectomy during Ladd’s procedure.

**Conclusion:**

It is difficult to implement a modification in a successful procedure like Ladd’s procedure. The majority of pediatric surgeons perform an appendectomy as part of its original description. This study has identified gaps in the literature pertaining to analyze the outcomes of performing Ladd's procedure without an appendectomy which should be explored in future research.

**Supplementary Information:**

The online version contains supplementary material available at 10.1007/s00383-023-05437-7.

## Introduction

Gastrointestinal malformations during the embryonic stages of development consist of a multitude of clinical presentations. Common malformations of the GI tract include underdevelopment of the midgut, abdominal wall defects, persistence of embryonic structures, abnormal neural development of the gut wall and malformations of intestinal rotation and fixation. Malrotation occurs when there are defects in the physiologic rotation of the intestines. Various anatomic descriptions have been given to the malrotation variants, including incomplete rotation, atypical malrotation, mixed rotation, and malrotation variant [1].

Fibrous bands have been shown to attach the cecum to the retroperitoneum in this malformation. These fibrous bands known as Ladd’s bands were described by American surgeon William Edward Ladd in 1932 [2]. The management of malrotation was initially described by Ladd and it consists of detorsion of the volvulus if present, division of Ladd’s bands, widening of the mesenteric root, proper positioning of the small and large bowels, and a prophylactic appendectomy [3]. It is believed that an appendectomy is performed for 2 main reasons: the placement of the appendix in the left upper quadrant would make future diagnosis of acute appendicitis delayed or sometimes missed and the dissection of the Ladd’s bands may cause damage to the appendiceal vessels supplying the appendix [4].

This procedure has been established as the gold standard of surgical management of malrotation for almost a century. Whilst the medical field is constantly advancing from invasive to non-invasive forms of management to improve patient prognosis and reduce post-operative complications, there is a transition from an open Ladd’s procedure to a laparoscopic approach [5].

Considering the advancement of accessibility and availability of patient medical records, thorough clinical assessment, advanced diagnostic testing and surgical techniques; the future misdiagnosis of acute appendicitis and appendiceal vessel damage intra-operatively should be dramatically reduced.

In accordance with the aforementioned advancements, we are trying to challenge the prophylactic appendectomy as part of Ladd’s procedure to keep the vermiform appendix for its benefits to the human body that have been highlighted in the accessible literature.

## Methodology

The study consists of 2 components:

1. A Systematic review that is reported according to the Preferred Reporting Items for Systematic Reviews and Meta-Analyses (PRISMA) guidelines [6].
2. A survey that was sent by email to 168 pediatric surgeons. The questions in the survey were centered on whether a surgeon performs an appendectomy as part of the Ladd’s procedure or not, as well as their reasoning behind either choice.

## Search strategy

The literature was reviewed through PubMed (MEDLINE) search, The articles were searched using the following MeSH terms:

(“ladd s procedure” [All Fields]) OR (“ladd procedure”[All Fields]).

(((“ladd procedure” [All Fields]) OR (“ladd s procedure”[All Fields]))) AND (“appendectomy”[All Fields]).

The use of broad search terms was done to ensure adequate screening of articles as not all papers had appendectomy as part of the MeSH Term. Furthermore, an inclusion criteria was designed to further focus the search and retrieval process.

## Eligibility criteria

The studies included in this review were according to the search terms used and the fulfillment of the following criteria:

1. Articles published in English
2. Articles about Ladd's procedure and appendectomy
3. Studies with participants in the age group; birth till 18 years.
4. All study designs within the past 30 years time frame (1992–2022)
5. PubMed indexed articles.

## Study selection

Five authors independently screened for the titles and abstracts of studies that were compatible using the aforementioned eligibility criteria. After the initial screening, full-text extraction from electronic databases and review of the eligible articles was conducted by these authors.

## Data collection and analysis

A short survey about Ladd’s procedure technique was designed collaboratively between the five authors using Google Form and shared with pediatric surgeons via email. The survey was written in English and sent out to our sample size of 168 pediatric surgeons whose practices are based in the Middle East in 2 different countries, the UAE and Iraq and the responses were received and recorded. In this survey, surgeons were allowed to choose more than one reason as to why they would or would not perform an appendectomy as part of Ladd’s procedure (as shown in form 1. Additionally, they were given the option to state any alternative reasons that were not mentioned in the list. Other factors such as years of experience and type of practices were not collected as they fall beyond the scope of this study where the main objective of the survey was to supplement our literature review by providing an approximation of the prevalence for the current practice of Ladd’s procedure within these 2 countries in the Middle East.

The responses were de-identified and exported into a spreadsheet to allow for analysis, interpretation, and graphical representation.

No ethical approval was required in this literature review as it is based on studies that are readily available on electronic databases. The participating surgeons agreed to participate in the survey and provided consent for the use of their responses.

## Risk of bias

This study is prone to language and selection bias as only articles in English were screened. It is also susceptible to selection bias as the survey was shared with 168 pediatric surgeons in two countries. While this may be valid, we have attempted to minimize the selection bias by having sent them to a significant number of surgeons within our reach as well as having five authors independently screen the articles during the literature search.

## Results

### Literature search

**Fig. 1:**
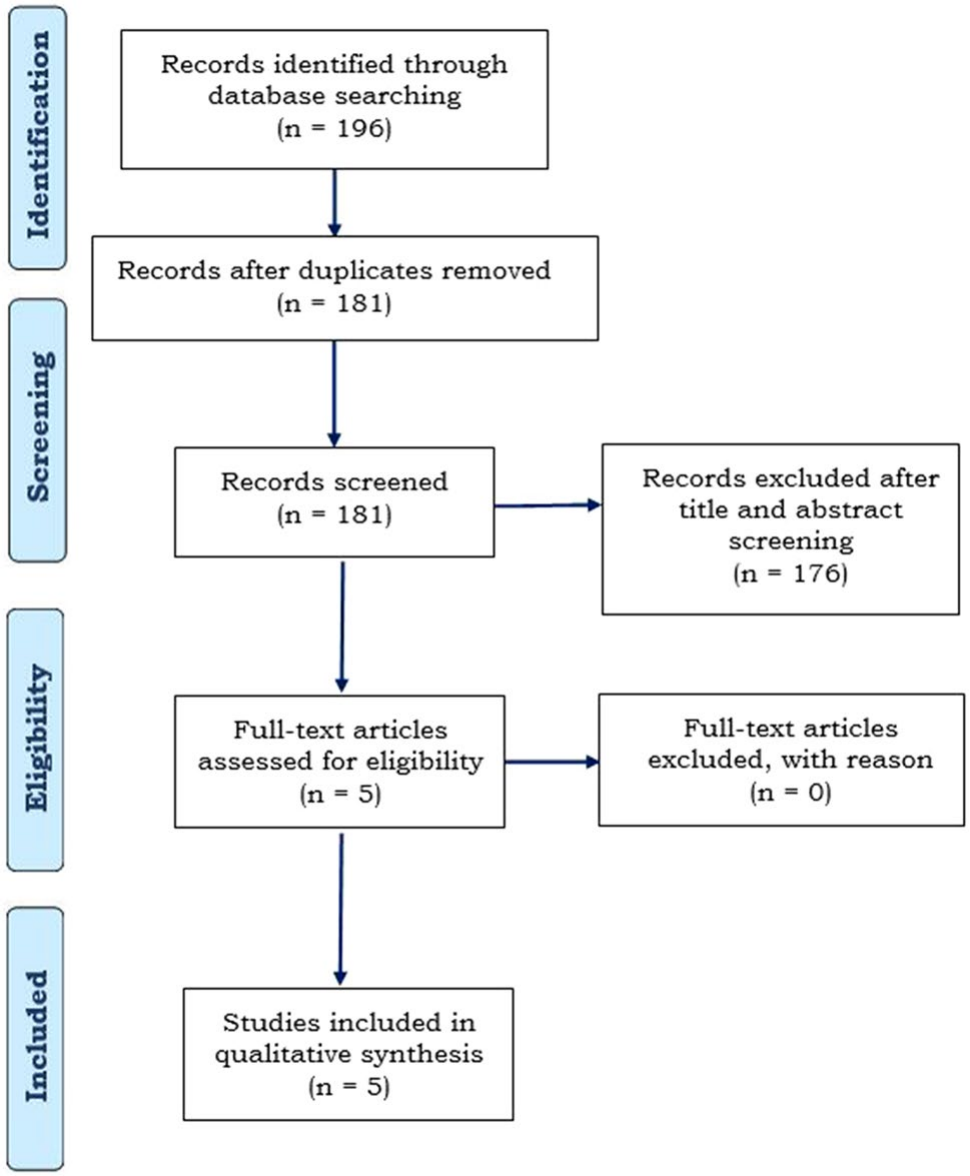
PRISMA flow diagram describing the literature review process

According to the search strategy used, 196 titles and abstracts were identified, duplicate studies were removed yielding 181 articles. Of the 181 titles and abstracts screened, 176 were excluded as they did not fulfill the eligibility criteria and 5 articles were compatible for full-text review. The PRISMA flow chart summarizing the data collection is depicted in Fig. 1.

### Search summary

Five articles were included in this review according to the inclusion criteria of the study, the essential data was extracted from these papers and depicted in Table 1.

**Table 1:** Summary of data extracted from the literature included in the review

Author/year	Study method	Number of participants with appendectomy	Number of participants without appendectomy	Outcome
Saberi et al. [9]	Retrospective review	1148	195	The majority of Ladd's procedures in the U.S. are being performed open, despite comparable outcomes following a laparoscopic approach. Readmission rates are similar with either approach, or the rate of redo Ladd's procedure is lower than previously reported
da Costa et al. [10]	Literature review	87	8	Laparoscopic Ladd's procedure with appendectomy and without cecopexy is the commonly practiced approach that is associated with minor complications
Svetanoff et al. [11]	Retrospective review	Laparoscopic Ladd’s: 14 Open Ladd’s: 10	Laparoscopic Ladd’s: 2 Open Ladd’s :12	Intraoperative characteristics were also similar between laparoscopic and open groups, respectively, in terms of operative time, performance of an appendectomy
Arnaud et al. [5]	Retrospective review	7	58	Laparoscopic Ladd's procedures is feasible, may reduce the risk of ashesive obstruction. A low threshod for conversion is important.
Kinlin et al. [12]	Cross-sectional survey	NA	NA	Only 1/8 who performed a LL as a standard approach routinely performed an appendectomy

*NA* Not available

The first study published by Saberi et al. in 2022 [7] comparing the outcomes of the Ladd’s procedure in terms of the surgical approach had a sample size of 1343 patients of which 85% underwent an appendectomy and 15% did not (195 patients) without providing clinical reasoning as to why they were done.

The second study published by da Costa et al. in 2021 [8] comparing laparoscopic and open Ladd’s procedure for malrotation in newborns and infants stated that appendectomy was not performed for 16 patients out of 99, without providing further information.

The third study published by Svetanoff et al. in 2020 [9] regarding the laparoscopic Ladd’s procedure for the management of malrotation and volvulus highlighted the Ladd’s technique and stated that the appendix is either removed or inverted. Furthermore from the sample size in the laparoscopic group 2 patients of the 16 did not undergo an appendectomy while in the open technique group 12 out of 22 patients did not undergo an appendectomy.

The fourth study published by Arnaud et al. in 2019 [3] addressing the fact that there is controversy in terms of the Ladd’s procedure approach stated that some surgeons perform a prophylactic appendectomy and/or cecopexy which was not present in the original description of the Ladd’s procedure by William Edwards Ladd. Moreover, these maneuvers have been abandoned by several centers in an effort to attempt to avoid the associated complications. They stated that out of 65 patients, only 7 had an appendectomy and the remainder did not.

The fifth study published by Kinlin et al. in 2017 [10] which was a study centered around the surgical management of malrotation based on a survey that involved the Canadian association of pediatric surgeons. The paper stated that while Ladd’s procedure with appendectomy has been a part of the procedure for decades, 27.6% of their respondents stated never or only sometimes removing the appendix. No further details about the reason behind each approach.

### Data from the survey

The survey was sent out to 168 pediatric surgeons. A total of 102 responses (60% response rate) were received. Ninety pediatric surgeons stated performing an appendectomy as part of the Ladd’s procedure (88%) while the remaining ten (12%) are not performing appendectomy.

**Fig. 2:**
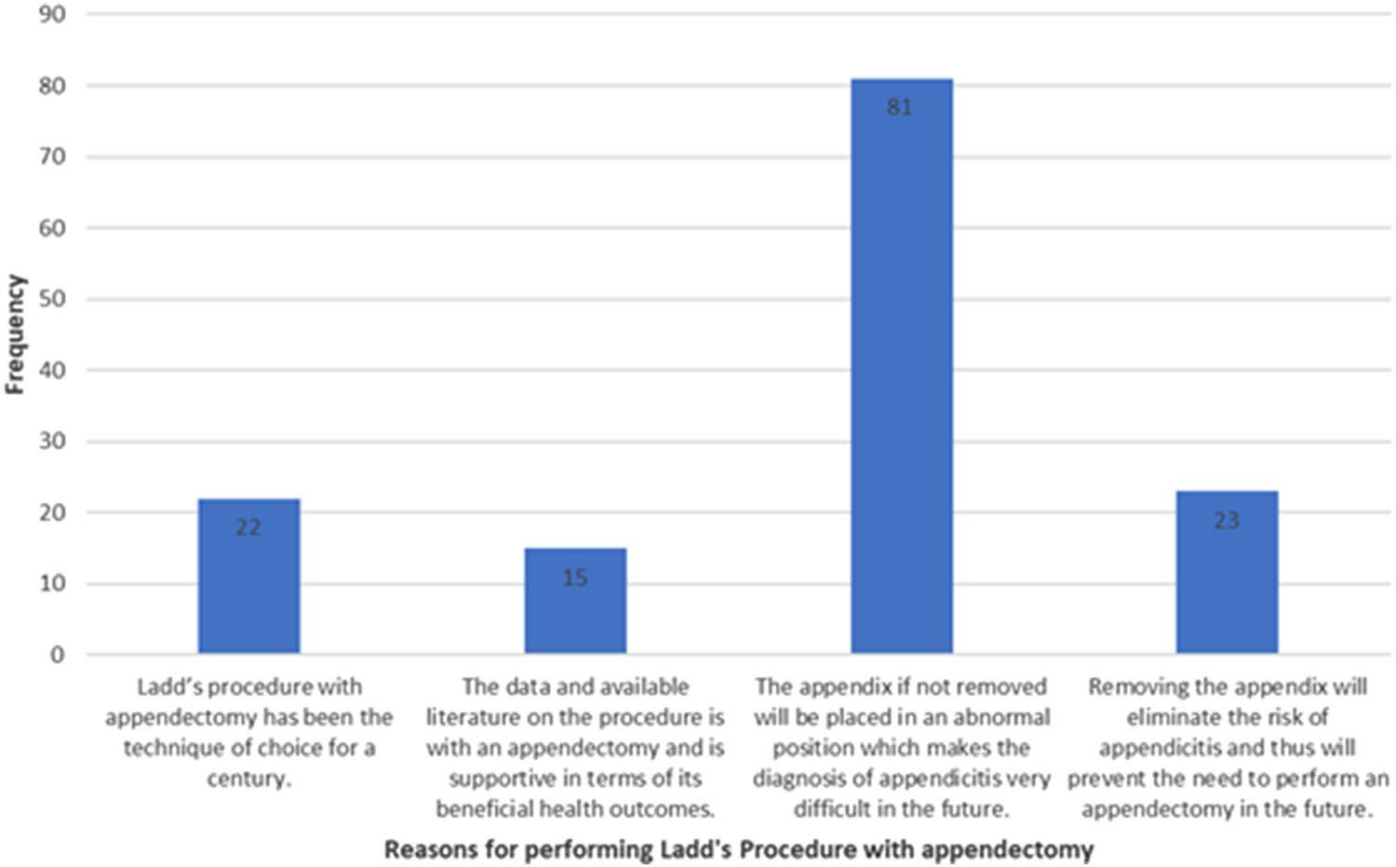
Reasons for performing appendectomy

Of the ninety responses received where surgeons stated performing an appendectomy; 81 surgeons chose the reason: “The appendix if not removed will be placed in an abnormal position which makes the diagnosis of appendicitis very difficult in the future”. Each surgeon was able to choose more than one option. Other reasons and their frequencies are detailed in Fig. 2.

**Fig. 3:**
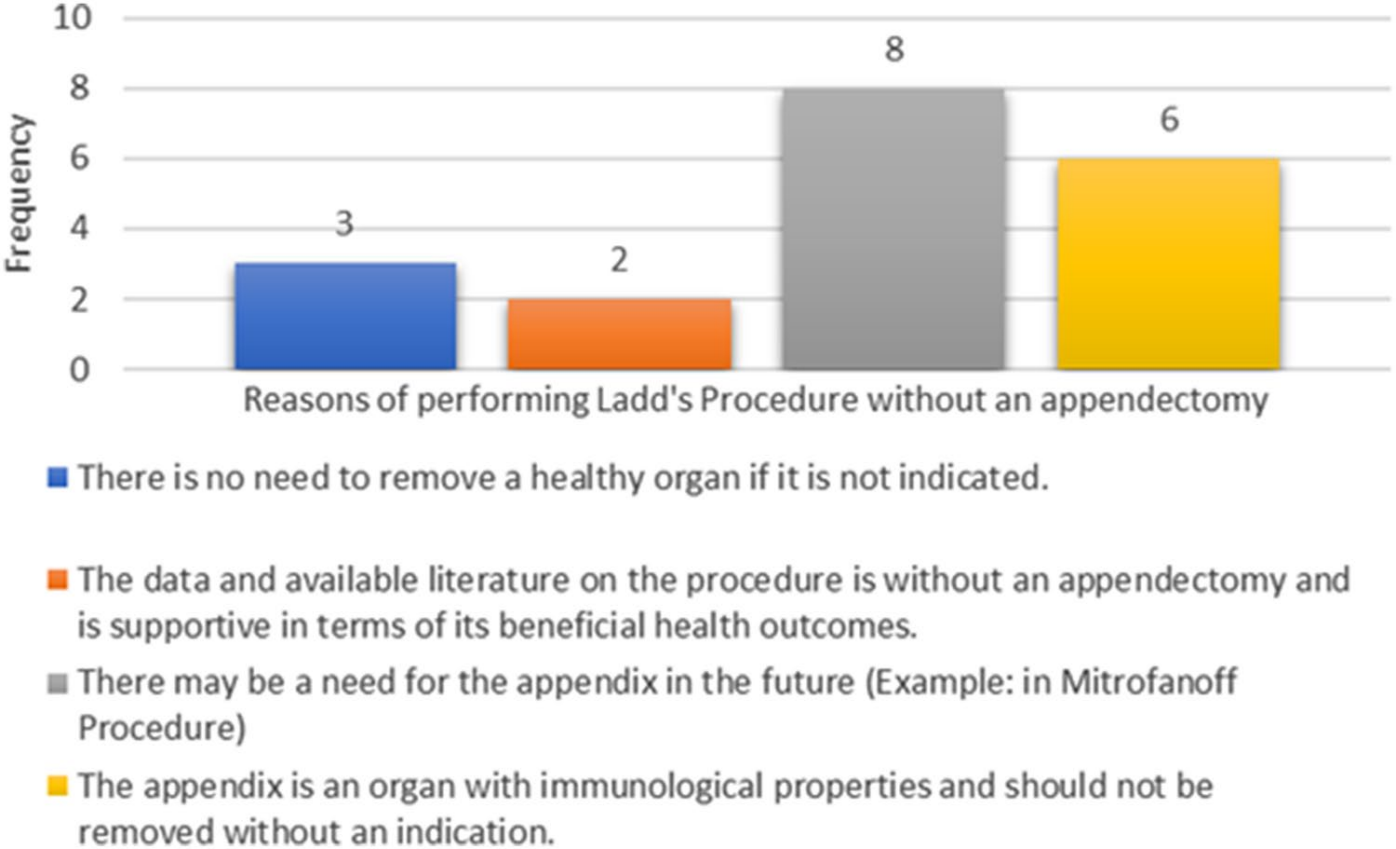
Reasons for not performing appendectomy

Of the 12 responses received where surgeons stated that they are not performing appendectomy, eight surgeons chose the following reason: “There may be a need for the appendix in the future (Example: in Mitrofanoff Procedure)”. Another reason: “The appendix is an organ with immunological properties and should not be removed without an indication” had been chosen by 6 surgeons. Each surgeon was able to choose more than one option. These results are depicted in Fig. 3.

## Discussion

The controversy regarding performing the Ladd’s procedure with or without an appendectomy remains unclear. This is partly attributed to the lack of evidence and available literature in assessing the benefits and risks of performing the procedure without an appendectomy. The results that we have yielded from our literature review correlate with the data that we have collected from our survey which states that appendectomies have been conducted in Ladd’s procedures for the past century and are still being conducted till the present day.

While there was no single study that analyzed the risks and benefits of performing Ladd’s procedure without an appendectomy, some of the aforementioned studies stated that there were cases of Ladd’s procedure performed without an appendectomy [3, 7, 8, 9, 10]. However, there was no mention of the clinical reasoning nor is there any available data on long term follow-up of these patients.

The survey of the fifth study [10] was sent out to 150 pediatric surgeons of the Canadian association and the 52 responses were then assessed. The study concluded that 70.2% of the surgeons always remove the appendix during the Ladd’s procedure. In comparison, the survey that was conducted as part of the current study was sent out to 168 pediatric surgeons, with 102 responses received back concluded that 90% of the surgeons perform an appendectomy as part of Ladd’s procedure. In our study each surgeon was allowed to give a reason or more to support their choice. The risk of future misdiagnosis of acute appendicitis was the main reason behind the decision of appendectomy (82 out of 90). On the other hand, 8 of the 12 surgeons are not doing appendectomy because of the potential need of future utilization of the appendix while 6 of them do not want to remove the appendix because of its immunological properties.

The roles of the appendix and its benefits to the human body had been discussed widely in the last 20 years and hence surgeons changed their approach from removing it as prophylaxis against appendicitis to keeping it for possible benefits and potential future use. A well-established utilization of the appendix is as a surgical conduit in the Mitrofanoff procedure and as an appendicostomy in the antegrade continence enema [11]. Another potential use of the appendix is for organ augmentation which has been studied in a rabbit model for augmentation cystoplasty [12].

It is hypothesized that the appendix is a reservoir of beneficial microbiota, as well as the significant benefit of the presence of lymphoid tissue (gut-associated lymphoid tissue) in the appendiceal mucosa [13, 14]. It has been proven that the removal of the appendix contributes to the alteration of the gut microbiota into a less diverse microbiome [15] and consequently increasing the potential risk of developing diseases, such as gastrointestinal cancer, rheumatoid arthritis, sarcoidosis, Parkinson’s disease, pyogenic liver abscess, and gallstone formation [17, 18, 19, 20, 21]. Moreover, it serves as a stem cell reservoir in which mesenchymal stem cells have been identified and isolated from human vermiform appendices [22] as well as neural stem cells which can successfully be differentiated into functioning and mature enteric neurons [23].

In the study published by Kinlin et al. in 2017 [10], the majority of respondents stated that for stable patients, laparoscopic and open Ladd’s procedures were equivalent surgical methods. Similarly, the study published by Saberi et al. in 2022 [7] shows both methods yield comparable results including readmission rates. In the study published by Svetanoff et al. in 2020, several factors were studied including operating times, post-operative results and complications, all of which were equivalent in the laparoscopic and open Ladd’s procedure approach. There is a lack of literature related to superiority in reference to performing an appendectomy in the open vs laparoscopic approach.

Traditionally, laparotomy was the preferred surgical method of choice for operating on Ladd’s bands supposedly due to the beneficial effects of post-operative adhesions, as the rates of adhesion formation post-laparotomy ranged from 50 to 90%. [24] It was conventionally thought that the post-operative adhesions contributed to the retroperitoneal fixation of the small and large bowels. This viewpoint is unclear as pediatric patients who have undergone laparoscopic repair do well. [25, 26] it is now believed that the main process of repair in Ladd’s procedure is the widening of the affected mesentery which ultimately reduces the likelihood of volvulus. This proves that adhesion formation may actually be more harmful than previously thought if it re-narrows the base of the mesentery. [25] This, alongside with decreased post-operative pain, shorter hospital stay, and better cosmetic outcomes shows the superiority of laparoscopic repairs over laparotomy.

Overall, this literature search further emphasizes that the topic of performing an appendectomy with the Ladd’s procedure remains understudied and calls for further research. It is a real challenge to accept modifying a procedure that proved to be successful for a century. A multicenter study with a significant sample size is suggested to provide more evidence into this topic.

## Conclusion

The majority of pediatric surgeons perform an appendectomy as part of the Ladd’s procedure which corresponds to the current available literature. The evidence and available literature that assesses the benefits and risks of not performing an appendectomy with Ladd’s procedure are not yet firmly established. There is a need for a multicenter study with significant sample size to strengthen the evidence and making the modification of Ladd’s procedure more acceptable worldwide.

### Supplementary Information

Below is the link to the electronic supplementary material.

Supplementary file1 (JPG 93 KB)

## Data Availability

The authors confirm that the data supporting the findings of this study are available within the article and its supplementary.
