# Supplementary figures and images for: Appendectomy as part of Ladd’s procedure: a systematic review and survey analysis
Source: Pediatr Surg Int. 2023 Apr 3;39(1):164. doi: 10.1007/s00383-023-05437-7 (PMC10070202; doi:10.1007/s00383-023-05437-7)

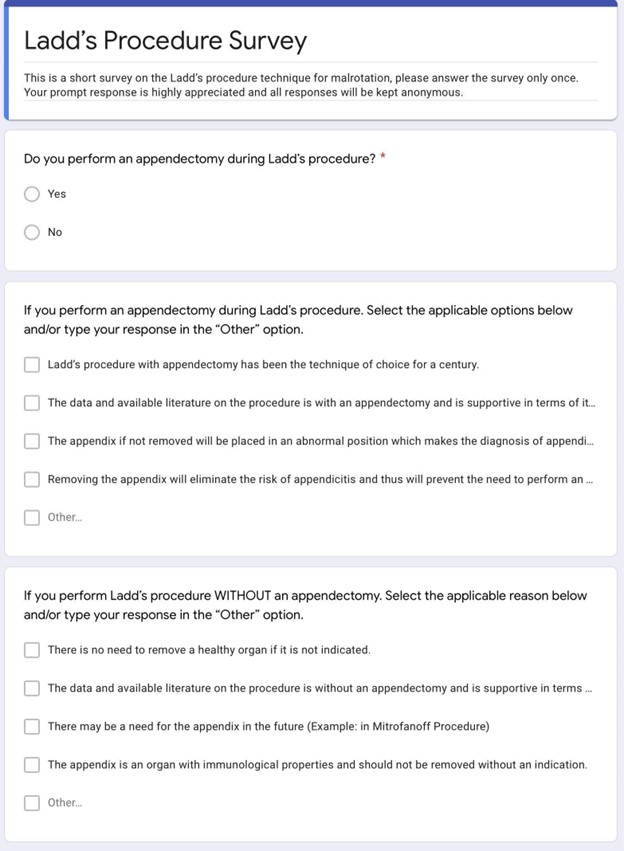

Supplement: Supplementary file 1 — Supplementary file1 (JPG 93 KB) [file 383_2023_5437_MOESM1_ESM.jpg]
